# Evening Primrose Extract Modulates TYMS Expression via SP1 Transcription Factor in Malignant Pleural Mesothelioma

**DOI:** 10.3390/cancers15205003

**Published:** 2023-10-16

**Authors:** Małgorzata Chmielewska-Kassassir, Katarzyna Sobierajska, Wojciech M. Ciszewski, Jakub Kryczka, Andrzej Zieleniak, Lucyna A. Wozniak

**Affiliations:** 1 Department of Structural Biology, Medical University of Lodz, Żeligowskiego 7/9, 90-752 Lodz, Poland; malgorzata.chmielewska-kassassir@umed.lodz.pl (M.C.-K.); andrzej.zieleniak@umed.lodz.pl (A.Z.); 2 Department of Molecular Cell Mechanisms, Medical University of Lodz, Mazowiecka 6/8, 92-215 Lodz, Poland; katarzyna.sobierajska@umed.lodz.pl (K.S.); wojciech.ciszewski@umed.lodz.pl (W.M.C.); 3 Institute of Medical Biology, Polish Academy of Sciences, Lodowa 106, 93-232 Lodz, Poland; jkryczka@cbm.pan.pl

**Keywords:** malignant pleural mesothelioma, evening primrose extract, thymidylate synthase, specificity protein 1 nuclear factor, epithelial-to-mesenchymal transition

## Abstract

**Simple Summary:**

Malignant pleural mesothelioma belongs to the aggressive tumor of pleura commonly recognized at the advanced, chemoresistant stage. Thymidylate synthase (TYMS) is a critical therapeutic target because it determines the susceptibility to folate-related drug treatment. We have previously reported that a natural extract isolated from defatted evening primrose seeds (EPE) reduces cell proliferation and the invasiveness of MPM cells. Therefore, we aimed to expound the mechanism of EPE in downregulating TYMS expression. Our bioinformatic analysis of an MPM patient genes data set confirmed the correlation of overexpressed TYMS with epithelial-to-mesenchymal transition biomarkers. Mechanistic studies of the culture cell have provided evidence for the direct effect of EPE compounds on the transcriptional activity of *tyms* promoter to its transcription factor, specificity protein 1. EPE mixture is a complex intervention related to the occupancy of SP1 motifs, blocking TYMS expression and triggering invasiveness of MPM cells.

**Abstract:**

Purpose: To determine the mechanism of EPE in downregulating TYMS in MPM cancer. Methods: The TYMS mRNA expression with epithelial-to-mesenchymal transition biomarkers and nuclear factor SP1 was assessed using the GEO database in a data set of MPM patients (GSE51024). Invasive MPM cell lines were in vitro models for the investigation of TYMS expression after EPE treatment. The *tyms* promoter SP1 binding sequences were determined using Genomatix v 3.4 software Electrophoretic mobility shift and dual-luciferase reporter assays revealed specific SP1 motifs in the interaction of EPE and reference compounds. Chromatin immunoprecipitation and Re-ChIP were used for the co-occupancy study. Results: In MPM patients, a positive correlation of overexpressed TYMS with mesenchymal TWIST1, FN1 and N-cadherin was observed. EPE and its major components, gallic and ellagic acid (GA and EA, respectively), downregulated TYMS in invasive MPM cells by interacting with particular SP1 motifs on the *tyms* promoter. The luciferase constructs confirmed the occupation of two SP1 regulatory regions critical for the promotion of TYMS expression. Both EPE and reference standards influenced SP1 translocation into the nucleus. Conclusion: EPE components reduced TYMS expression by occupation of SP1 motifs on the *tyms* promoter and reversed the EMT phenotype of invasive MPM cells. Further in-depth analysis of the molecular docking of polyphenol compounds with SP1 regulatory motifs is required.

## 1. Introduction

Malignant pleural mesothelioma (MPM), an aggressive neoplasm that develops for many years without manifesting symptoms, is characterized by poor prognostic outcomes and has a 5-year survival that is noted in only 10% of patients [1]. The chemotherapy recommended for MPM, commonly based on a multitargeted antimetabolite approach against folic acid metabolism-related enzymes, prolongs the survival by only 2.8 months [2].

The reason for the resistance to folate-related antimetabolites is seen in thymidylate synthase (TYMS), whose overexpression has been observed in several solid tumors, including tumors associated with breast cancer, colorectal cancer and mesothelioma [3,4,5]. TYMS upregulation correlates with the invasive phenotype of cancer cells, corresponding with the epithelial-to-mesenchymal transition (EMT) process that is manifested in metastasis [4].

Polyphenols are an important group of plant metabolites as their consumption reduces the risk of cancer development [6]. The beneficial antitumor effect of these phytochemicals, both as individual compounds and their combinations, is reflected in the elevation of cancer cell death via the stimulation of apoptotic signaling and the inhibition of proliferating signals [7,8,9]. A modulatory effect on the EMT process is also attributed to their role [10,11]. Evening primrose extracts (EPE), rich in polyphenols, have been isolated from postindustrial defatted *Oenothera paradoxa* seeds and revealed potential anti-cancer properties [9,12,13,14]. It is known that EPE constituents might inhibit the metastatic abilities of cancer cells by downregulating metalloproteinase expression and secretion [15,16]. We have previously reported that MPM cell lines with a more invasive phenotype are more susceptible to EPE treatment [14] and revealed decreased motility. Recently, we have demonstrated that EPE regulates the metabolic switch in EMT and reduces the invasive abilities of colorectal cancer (CRC) cells [9]. In addition, we correlated the inhibitory effect of EPE with the reduction in TYMS level, and observed a slightly increased sensitivity of CRC cells to the chemotherapeutic agent, 5-fluorouracil. These data strongly indicate that the natural plant EP extract can be a supportive treatment strategy for invasive malignancies.

Considering the critical role of TYMS in cancer cell growth, proliferation and metastasis, for this paper we established, at the molecular level, the inhibitory effect of EPE on TYMS downregulation in mesothelioma cancer cells. The human *tyms* promoter region was well characterized by Horie et al. a long time ago [17], when they determined the regulatory fragments essential for TYMS expression, in which specificity protein-1 nuclear factor (SP1) motifs were present. SP1 is a multifunctional zinc-finger transcription factor that regulates cellular processes, including the cell cycle and apoptosis. SP1 also contributes to the malignant phenotype by regulating oncogenes that influence tumor cell proliferation, invasion and metastasis [18]. Recent data have revealed that polyphenols and other phytochemicals are potential modulators of SP1 and SP1-related genes [7]. Thus, in light of the widespread application of therapeutic strategies based on an anti-folate treatment against TYMS, such as pemetrexed in MPM [19] or 5-FU in CRC [9], and the unprecedented role of natural compounds in tumor prevention and suppression, we investigated the molecular mechanisms of TYMS expression regulation upon EPE treatment. In detail, we focused mainly on the potency of the SP1 motifs on TYMS expression by the SP1 nuclear factor activity modulated in response to polyphenol-rich extract obtained from the defatted evening primrose seeds. In addition, we verified the effect of selected, single components of EPE on TYMS transcription regulation and eliminated the individual application of EPE compounds, indicating the benefits of EPE mixture use against MPM cancer.

## 2. Materials and Methods

### 2.1. Extract Preparation and Compound Analysis

The evening primrose extract (EPE) was obtained from postindustrial defatted evening primrose (*Oenothera paradoxa*, Hudziok) seeds that have been chemically characterized previously [14]. The mixture contained polyphenol compounds in approximately 21% of the dried extract (208.24 ± 8.29 mg per gram), with a predominant content of phenolic acids: gallic acid (GA) and ellagic acid (EA), which compose 24.7% and 37.3% of the total determined polyphenols, respectively [14].

### 2.2. Bioinformatic Analysis of MPM Patient Samples

Microarray profiles of surgically resected MPM tumors and paired non-tumor adjacent tissue (serving as controls) were acquired from the public Gene Expression Omnibus (GEO) database (National Center for Biotechnology Information (NCBI), U.S. National Library of Medicine 8600 Rockville Pike, Bethesda MD, 20894 USA, dataset: GSE51024T (Affymetrix Human Genome U133 Plus 2.0 platform)). The dataset consisted of 96 samples divided into 2 groups: cancer samples (named: Cancer tissue) *n* = 54, and non-malignant normal tissue surrounding mesothelioma (named: Adjacent tissue) *n* = 42. Obtained transcriptomic data, consisting of mRNA levels of the genes of interest, were analyzed as described earlier [20].

### 2.3. Cell Culture and Treatment Conditions

Human malignant pleural mesothelioma cell lines, sarcomatoid JU77, biphasic MSTO-H211 and pleural effusion metastatic NCI-H28, were donated by Prof Peter Szlosarek from Queen Mary University of London, UK. Cells were grown in previously reported conditions [14] and treated at a non-cytotoxic concentration of 200 µg/mL of EPE, corresponding to 41.6 µg/mL gallic acid equivalents (GAE). In the case of single polyphenols—gallic acid (GA) and ellagic acid (EA)—the concentration corresponded with GA and EA content in EPE preparation—24.7% and 37.3% of total polyphenols present in EPE preparation, respectively—and were added at concentrations of 15.4 µg/mL EA and 10.3 µg/mL GA [14]. Both were also studied at a maximal 41.6 µg/mL GAE dosage, equal to the total TPC present in the EPE mixture as in the case of urolithin A—an EA metabolite. The final ethanol concentration did not exceed 0.1% in the culture medium.

### 2.4. Quantitative Real-Time PCR

Total RNA was isolated using RNAzol reagent (Invitrogen, Carlsbad, CA). First-strand cDNA was synthesized from 4 µg of RNA using the RevertAid H Minus First Strand cDNA Synthesis Kit according to the manufacturer’s instructions. The sequences of specific human *TYMS*, *SP1* and *GAPDH* (with glyceraldehyde 3-phosphate dehydrogenase, as internal control) primer pairs were designed based on Ensemble Genome Browser transcripts with RT-qPCR amplicon lengths indicated in Table 1. Relative gene expression was determined using Maxima SYBR Green/ROX qPCR Master Mix (2×) (Thermo Scientific™, Waltham, MA, USA) and a standard protocol on a 7500 Fast Real-Time PCR system. GAPDH was used to normalize the target transcripts. The specificity of the product was assessed from the melting curve analysis. The fold change in target mRNA expression was estimated using the 2^−ΔΔCt^ quantification method.

### 2.5. Western Blot Analysis

Cells were harvested by scraping in M-PER lysis buffer (Rockford, IL, USA) containing proteinase and phosphatase inhibitor cocktail (Sigma-Aldrich, St. Louis, MO, USA), and precipitated protein concentrations of whole cell lysates were determined using a Pierce BCA Protein Assay Kit (Rockford, IL, USA). Twenty micrograms of total proteins were separated on SDS-PAGE (10% Tris-Glycine gel) and transferred to a nitrocellulose membrane (Bio-Rad) using a Mini-PROTEAN Bio-Rad System and further proceeded as previously described [21]. The blocked membrane was incubated with the primary antibodies, monoclonal anti-TYMS, clone TS106 (#MAB4130) Merck Millipore^®^ (Billerica, MA, USA), (1:2000), or anti-SP1 (#sc-17824) (Santa Cruz Biotechnology, Dallas, TX, USA), in TBST overnight at 4 °C and further incubated with the secondary goat anti-mouse antibody conjugated with horseradish peroxidase (#P0450) (Dako, Ely, UK). The chemiluminescence detection was processed using SuperSignal™ West Pico PLUS Chemiluminescence Substrate Kit (Rockford, IL, USA), and membranes were fixed on Kodak BioMax Light Film (Eastman Kodak, New York, NY, USA). The developed films were scanned, and the visualized bands were quantitated using ImageJ v 1.47 software. Subsequently, the membrane was incubated with Restore™ PLUS Western Blot Stripping Buffer (Rockford, IL, USA). Then, it was treated as described above in order to detect and quantify the GAPDH level for protein normalization using an anti-GAPDH antibody (#sc-166574) (Santa Cruz Biotechnology, Dallas, TX, USA). According to the manufacturer’s instructions, nuclear extraction material was obtained from cultured cells using NE-PER™ Nuclear and Cytoplasmic Extraction Reagents (Rockford, IL, USA). The SP1 protein level in nuclear fraction was determined as described above with normalization to histone H3 (His-3) expression using an anti-His-3 antibody (#sc-8036) (Santa Cruz Biotechnology, Dallas, TX, USA). For EMT markers we used the following antibodies, Snail (#C15D3), ZEB1 (D80D3), N-cadherin (D4R1H), vimentin (D21H3), E-cadherin (24E10), occludin (E6B4R) from Cell Signaling Technology in 1:1000 concentration, were employed.

### 2.6. Confocal Microscopy

MPM cells (1 × 10^5^), fixed for 20 min at room temperature with 4% formaldehyde in PHEM buffer, at a pH of 6.9 (60 mM PIPES, 25 mM HEPES, 10 mM EGTA and 4 mM MgCl_2_) and containing cOmplete™, Mini, EDTA-free Protease Inhibitor Cocktail (Roche, Basilea, Switzerland) were further processed as previously described [21]. SP1 localization within a cell was determined using specific anti-SP1 antibodies and then incubated with the secondary goat anti-mouse polyclonal antibodies conjugated with Alexa448 Thermo Scientific (Rockford, IL, USA) for 2 h at room temperature. Nucleus counterstaining was performed using 4’,6-diamidino-2-phenylindole (DAPI) dye under a Leica TCS SP8 confocal laser microscope system with a high-resolution objective (63×/1.4; oil). A series of single optical sections were collected every 0.2 μm. Quantification of protein localization in the regions of interest were analyzed using ImageJ v 1.47 software.

### 2.7. Electrophoretic Mobility Shift Assay

As previously described, EMSA experiments were conducted using the GelShift Chemiluminescent EMSA Kit Promega (Madison, WI, USA) [22]. The nuclear extracts were prepared as described in the Western blot method section. The applied oligonucleotide probe sequences were as follows: positive control with sequence homologous to the fragment upstream of the site of the transcription initiation of *tyms*, and a non-specific negative control deprived of the *tyms* binding motifs. For the competitor test, an unlabeled oligonucleotide probe was used in excess. Monoclonal antibodies against SP1 were used for supershift mobility monitoring. After sample separation in a 10% polyacrylamide gel and transfer onto a nitrocellulose membrane, DNA was hybridized to the membranes under UV light. The biotin-labeled probes were detected using streptavidin conjugated with horseradish peroxidase. The developed chemiluminescence process was visualized on an HP Scanjet G4050 scanner (Hewlett Packard, Palo Alto, CA, USA). Protein bands were analyzed using ImageJ v 1.47 software.

### 2.8. Luciferase Reporter Assay

A luciferase reporter assay was performed using Dual-Luciferase Reporter Assay (Promega, Madison, WI, USA) according to the previously described method [22]. The *tyms* promoter region was amplified by PCR, forward 5′-3′ CGCGCGGAAGGGGTCCTG, and reverse 5′-3′ GGAGGATGTGTTGGATCTGC-3′, and cloned into the pGL4 vector (Promega). All cell lines were transfected with the pGL4/promoter construct with the parallel plasmid pRTK containing luciferase dedicated for normalization of transfection efficiency. After 48 h, the total activity of luciferase emitted upon SP1 binding was measured using the Dual-Luciferase Reporter Assay System according to the manufacturer’s instructions.

### 2.9. Point Mutation Introduction

The point mutations were introduced using the GeneTailor Site-Directed Mutagenesis System, as indicated previously [22] and with the appropriate primers: forward 5′-3′-TCTCTAGAGCGGGGACGTCCGCGACCCCGCCGA and reverse 5′-3′-AGCCTCGACGGCGCGAATTCCGATCGTAA

### 2.10. Chromatin Immunoprecipitation (ChIP) and Re-ChIP Assay

Chromatin immunoprecipitation (ChIP) analysis was performed using the Pierce™ Agarose ChIP Kit according to the manufacturer’s instructions as previously described [22]. Next, Re-ChIP analysis was conducted according to the previous methodology. The final homogenate was re-incubated with anti-SP1 antibodies and the PCR products (forward 5′-3-TCTACTCCCTCCCTCCCTTC-3; reverse 5′-3′-CTCGAGTCTGCCAGTGACAA) were separated in 7% polyacrylamide gels. The bands were stained with ethidium bromide, visualized by UV light, and documented by the GelDoc EQ gel documentation system (Bio-Rad, Hercules, CA, USA). The results of a Re-ChIP experiment of co-occupancy were calculated as previously described [23].

### 2.11. Statistical Analysis

The statistical significance of the differences between experiments was determined by the Student’s *t*-test or by ANOVA followed by Tukey’s test. Data distribution in all cohorts was analyzed using the Shapiro–Wilk normality test. For not normally distributed cohorts, the Mann–Whitney U-Test was used instead of the Student’s t-test. Gene expression correlation was calculated and presented using JASP 0.14.1.0 software and Pearson’s correlation module. The significance level was set at *p* = 0.05. The relationship between the invasion ability and TYMS protein level was determined by linear regression analysis.

All analyses were performed using GraphPad Prism version 9.3 software (GraphPad Inc., San Diego, CA, USA) based on at least three independent experiments. The data are presented as the means ± standard deviations unless otherwise stated.

## 3. Results

### 3.1. EMT Regulator Expression Is Modulated in MPM Patients

**Figure 1:**
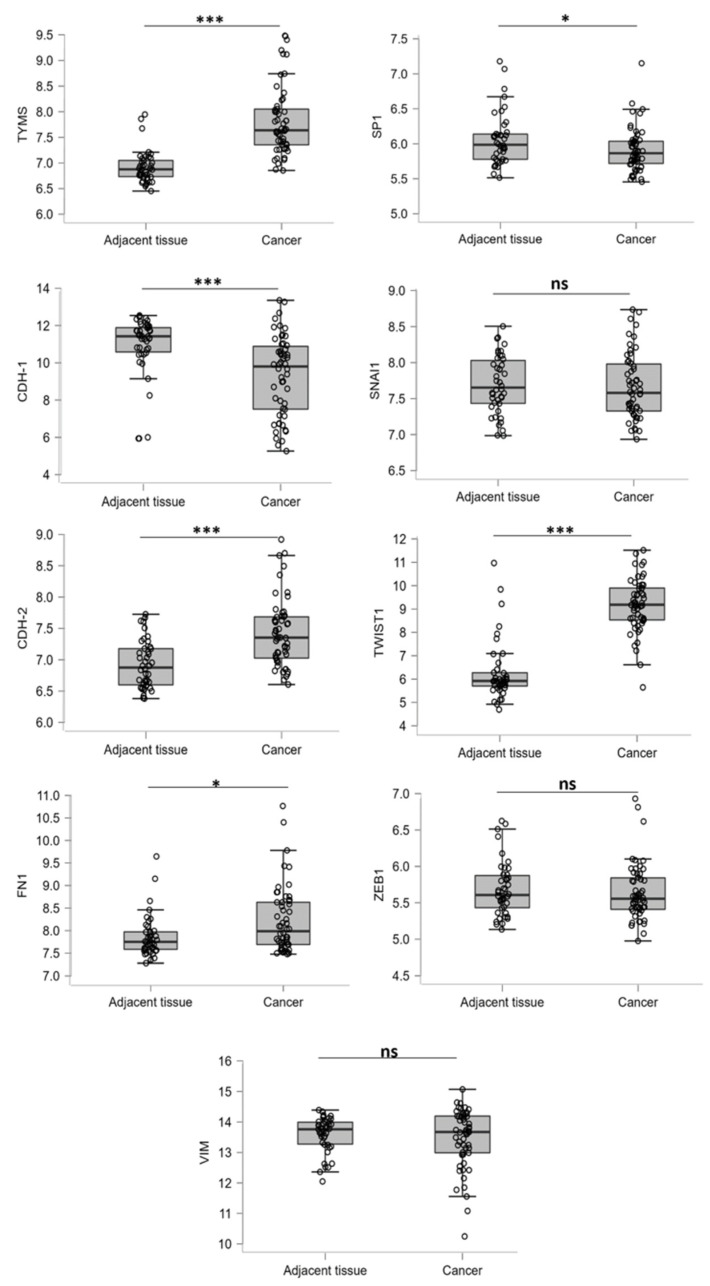
The mRNA expression levels in cancer and paired adjacent normal tissues in MPM patients. Normal patient cohorts of *TYMS*, *SP1*, *FN1*, *CDH-1*, *SNAl1*, *VIM*, *CDH-2*, *TWIST1* and *ZEB*. Data were obtained using the Gene Expression Omnibus (GEO) NCBI database (data set GSE51024). ns—non-significant; * *p* < 0.05 and *** *p* < 0.001.

MPM is classified according to cell origin into three histological subtypes: epithelioid, sarcomatoid, and biphasic [24] which in turn reflect distinct epithelia–mesenchymal transition marker expression profiles associated with a miscellaneous predictive value [25]. The critical role has been assigned to Twist (TWIST1) and ZEB-1, whose overexpression has been associated with a poor prognosis and worse survival rate [26,27]. Herein, we ascertained the levels of some epithelial and mesenchymal markers, particularly transcription factors (ZEB-1, Twist, Snail) and *tyms* promoter regulator SP1 with TYMS expression in the mesothelioma cancer and non-tumor adjacent tissues by bioinformatics analysis. We found upregulation of both *TYMS* and mesenchymal *TWIST1*, as well as N-cadherin (*CDH-2*) and filamin 1 (*FN1*) in primary tumors, compared with paired normal tissues (Figure 1). Vimentin (*VIM*), ZEB-1 and Snail (*SNAI1*) exhibited no significant differences. In contrast, epithelial cadherin (*CDH-1*) was downregulated in cancer tissues compared with control samples, strongly potentiating the transdifferentiation process in MPM cancer tissue (Figure 1).

**Figure 2:**
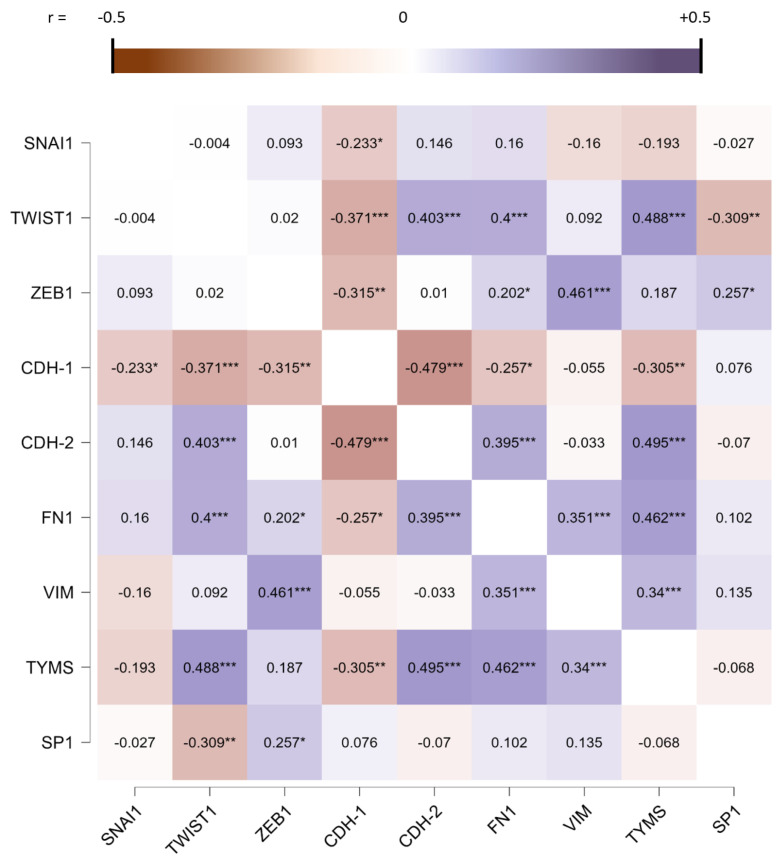
The Pearson correlation matrix of *SNAI1*, *TWIST1*, *ZEB1*, *CDH-1*, *CDH-2*, *FN1*, *VIM*, *TYMS* and *SP1*. * *p* < 0.05, ** *p* < 0.005, and *** *p* < 0.001.

The Pearson correlation matrix demonstrated a statistically significant positive correlation of *TYMS* with the mesenchymal phenotype biomarkers *TWIST*, *FN1*, and *CDH-2* but not *ZEB1* and *VIM*, and a downregulation of *CDH-1* (Figure 2). Despite the indication of the invasive nature of MPM, we did not observe a statistically significant correlation for *SP1* except for the upregulation of *TWIST1* (0.309).

### 3.2. EPE Reverse Epithelial-Mesenchymal Transition in MPM Cell Lines

**Figure 3:**
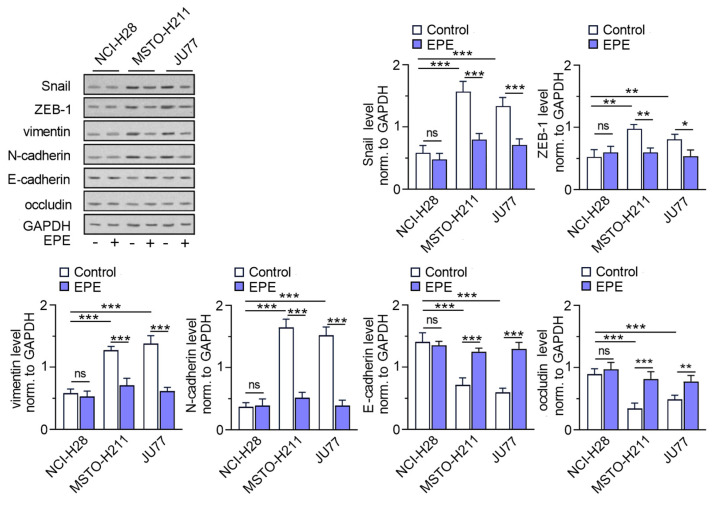
EPE modulates expression of EMT markers. MSTO-H211, JU77 and NCI-H28 cells were treated with EPE (200 µg/mL) for 48 h for protein extraction. Snail, ZEB1, vimentin, N-cadherin, E-cadherin and occludin protein levels were analyzed. Representative blots are shown. The graphs display the mean protein levels normalized to GAPDH ± SD. ns—non-significant, * *p* < 0.05, ** *p* < 0.005 and *** *p* < 0.001. The uncropped blots are shown in Supplementary File S1.

Then, we tested if the level of EMT markers accompanied the previously detected differences in the invasion ability observed in MPM cell lines (Figure 3). We have revealed that, characterized by higher invasion, JU77 and MSTO-H211 cells demonstrated higher levels of mesenchymal markers (vimentin, N-cadherin) and EMT transcription factors (Snail and ZEB1) in comparison with low invasive NCI-H28 cells. Additionally, the levels of epithelial proteins (E-cadherin and occludin) were correspondingly lower. Moreover, we were able to reveal that the EPE treatment caused the decreased EMT transcription factors and mesenchymal marker levels, while increasing the level of epithelial marker levels in JU77 and MSTO-H211 cells. Those processes were not detected in NCI-H28 cells.

### 3.3. The TYMS Expression Profile in MPM Cells Is Dependent on Its Invasion Ability and Downregulated by EPE Treatment

**Figure 4:**
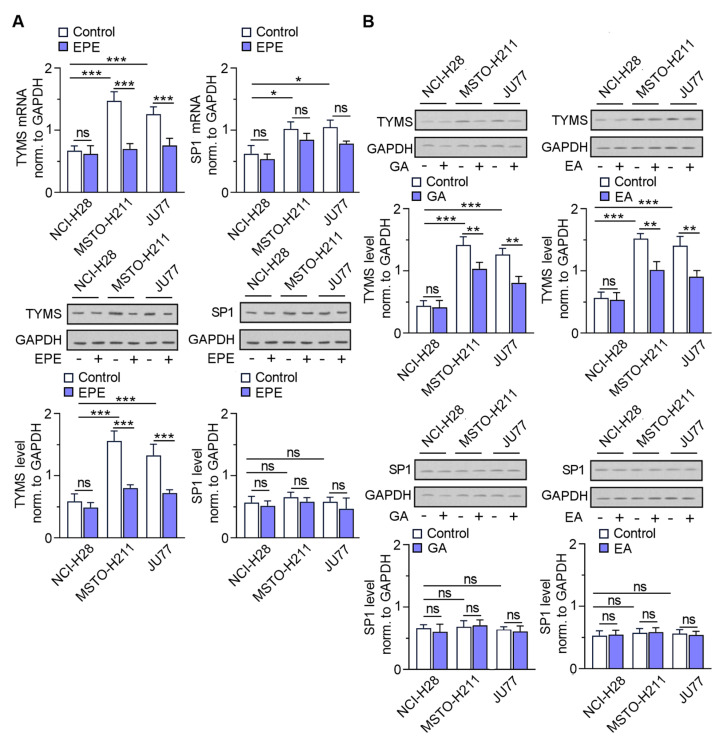

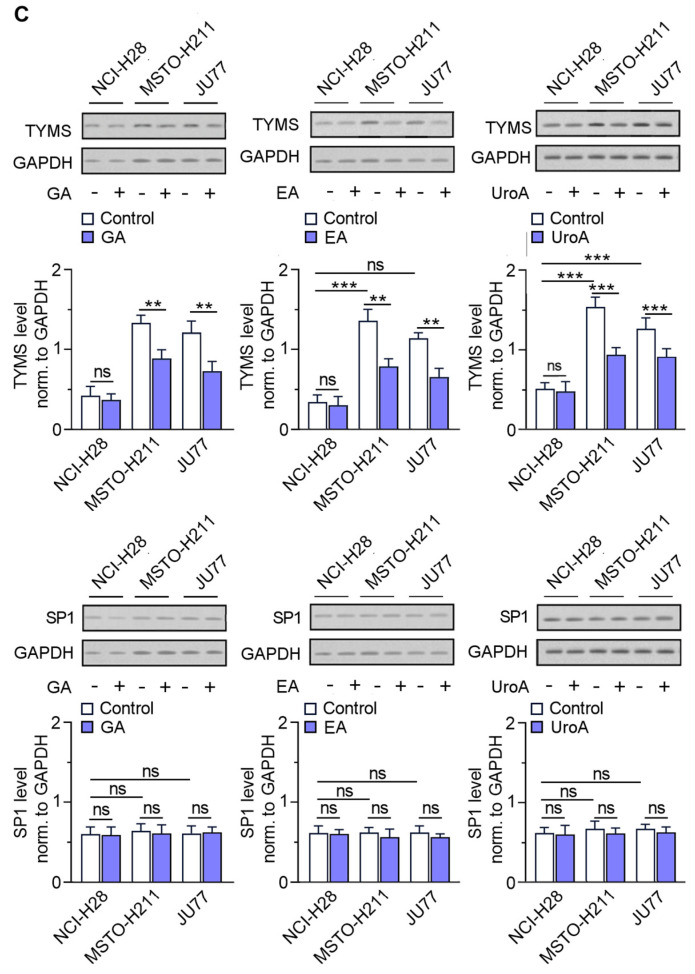
EPE downregulates TYMS expression in invasive MPM cells and does not modulate SP1 mRNA and protein levels. MSTO-H211, JU77 and NCI-H28 cells were treated with EPE (200 µg/mL) or reference compounds (15.4 µg/mL EA and 10.3 µg/mL GA, or 41.6 µg/mL GA, EA or UroA) for 24 h for RNA isolation and 48 h for protein extraction. (**A**) Relative *TYMS* and SP1 mRNA and protein levels normalized to GAPDH in EPE-treated and control MPM cells. (**B**) TYMS and SP1 protein level normalized to GAPDH upon GA and EA treatment at a concentration corresponding to % of total polyphenols in EPE. (**C**) TYMS and SP1 protein levels normalized to GAPDH upon GA, EA and UroA treatment at a concentration corresponding to the maximum TPC of the EPE mixture. Representative blots are shown. The graphs display the mean mRNA and protein levels normalized to GAPDH ± SD. ns—non-significant * *p* < 0.05, ** *p* < 0.005 and *** *p* < 0.001. The uncropped blots are shown in Supplementary File S1.

To confirm the clinical data of TYMS overexpression status in an in vitro model of the studied MPM cell lines, we further analyzed the mRNA expression and protein level of TYMS, and one of its regulators, SP1. Our culture studies revealed approximately a 1.5 times higher *TYMS* expression in JU77 and MSTO-H211 cells, previously reported as those with a high invasive phenotype [14], when compared with NCl-H28, characterized as similarly low invasive cells, at the transcription level (Figure 4A). The TYMS protein level in both cell lines demonstrates a 3.0-fold elevation over control cells (Figure 3A). Because we recently reported that treating colon cancer cells with EPE modulates their invasiveness in a TYMS-dependent manner [9], we checked our extract’s influence in the MPM cells model. Upon EPE treatment, we found that the expression of TYMS significantly decreased in EPE-sensitive MSTO-H211 and JU77 cells, previously reported as invasive subtypes [14], in contrast with NCl-H28 cells (Figure 4A). Surprisingly, observed no modulation of SP1 in any of the tested cell lines, neither for controls nor for EPE-treated cells (Figure 4A).

Because EPE preparation comprises a mixture of different polyphenols, we verified the parallel use of single compounds, gallic acid (GA) and ellagic acid (EA), that are present in large amounts in our extract, as well as EA metabolite–urolithin A (UroA) on TYMS protein level. We observed a weaker effect on TYMS level downregulation at both studied concentration variants where GA and EA were applied in a concentration corresponding to % content in the EPE preparation (EA 15.4 µg/mL and GA 10.3 µg/mL—Figure 4B) or the total polyphenols content present in EPE (41.6 µg/mL) in the cases of GA, EA and UroA (Figure 4C). Our Western blot study (Figure 4B,C) revealed that SP1 protein did not change significantly in any investigated MPM cells upon treatment with the desired compounds and at the tested concentrations variants. These observations indicate the beneficial application of a mixture of components, rather than individual EPE components alone, even at the highest polyphenol content present in EPE preparation. Additionally, based on our previous studies about the invasion ability of MPM cell lines [14], we calculated a correlation coefficient (R) between invasion ability and TYMS level that amounted to 0.84 (Figure S1).

### 3.4. SP1 Localization in Nucleus Is Significantly Reduced in the EPE-Treated Cells

**Figure 5:**
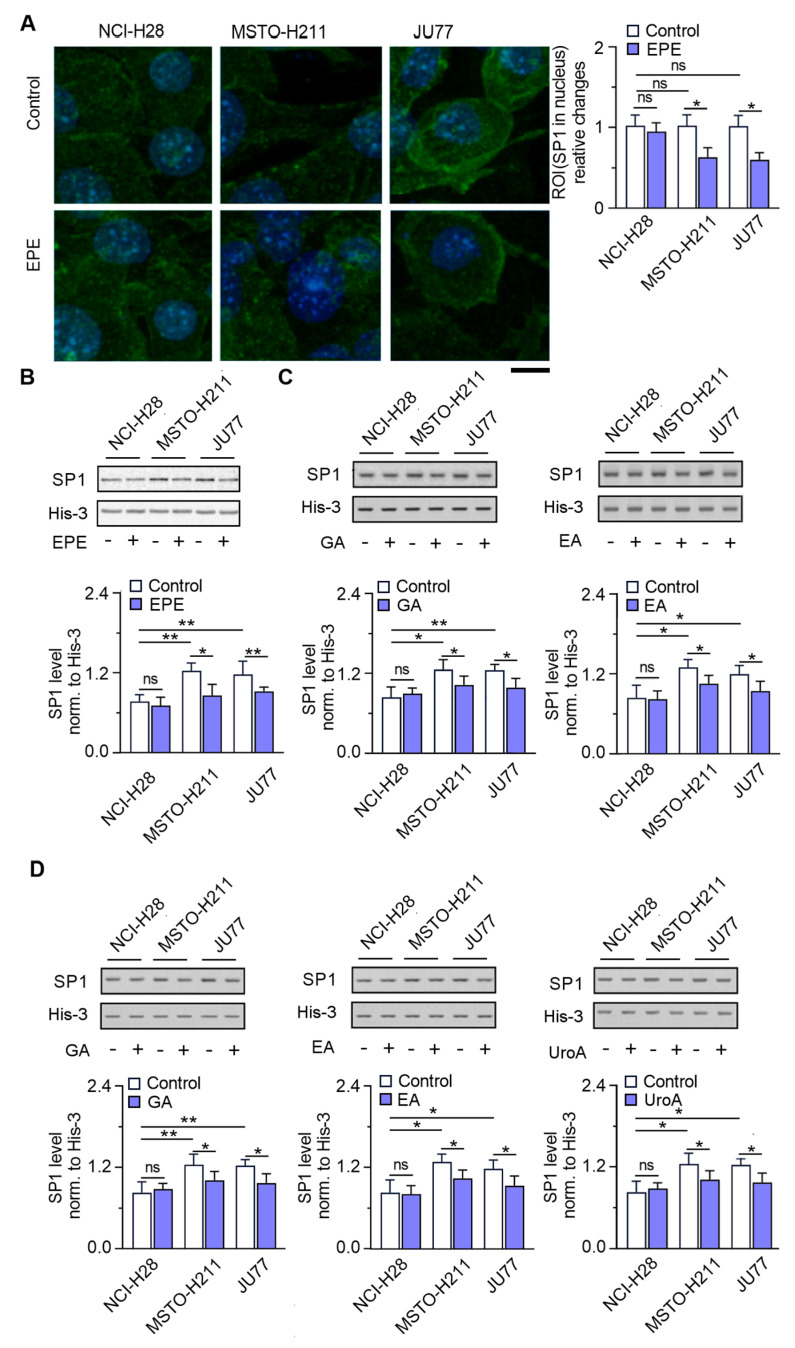
EPE modulates the localization of the SP1 nuclear factor in MPM cells. Before analysis, MPM cells were treated for 48 h with either EPE (200 µg/mL) or single polyphenolic compounds GA, EA, and UroA. (**A**) The localization of SP1 in the untreated control (upper panel) and EPE-treated (bottom panel) MPM cells. Representative images of SP1 localization in cellular compartments were determined using fluorescence microscopy with evaluated signals of the region of interest (ROI) in the nucleus of experimental MPM cells. The black bar represents a 50 µm length. (**B**) The relative SP1 protein level in the nuclear fraction after EPE treatment normalized to His-3 ± SD. (**C**) The SP1 protein level in the nuclear fraction after GA and EA treatment at a concentration corresponding to % content of TPC in EPE, 10.3 µg/mL and 15.4 µg/mL, respectively. (**D**) The SP1 protein level in the nuclear fraction after GA, EA and UroA treatment at a concentration corresponding to total TPC content—41.6 µg/mL. ns—non-significant, * *p* < 0.05 and ** *p* < 0.01. The uncropped blots are shown in Supplementary File S1.

Because we did not detect any changes in the SP1 expression (Figure 4A) in response to EPE, we decided to check its localization in EPE-treated mesothelioma cells. The nuclear SP1 accumulation was approximately 30% lower in the invasive EPE-treated MSTO-H211 and JU77 cells (Figure 5A), as revealed by the significantly reduced (37%, and 31% respectively) SP1 nuclear protein compared with untreated control cells. In contrast, we did not detect any changes in the SP1 translocation and protein level in NCI-H28 cells (Figure 5A,B). Administration of single GA and EA, both used at a concentration corresponding to their amount in EPE, 10.3 µg/mL and 15.4 µg/mL GAE, resulted in a weaker effect and lower SP1 levels of 19% and 20%, respectively, than those of the control untreated cells (Figure 5C). Similarly, intervention with standards at the concentration corresponding to total TPC in EPE (41.6 µg/mL) resulted in 25%, 21% and 24%, respectively for EA, GA and UroA, as well as reduced SP1 protein in invasive MPM cells compared with EPE-untreated cells (Figure 5D). For NCI-H28 cells, we did not observe any statistically significant changes.

### 3.5. Identification of Regulatory SP1 Motifs in the Tyms Promoter Region

**Figure 6:**
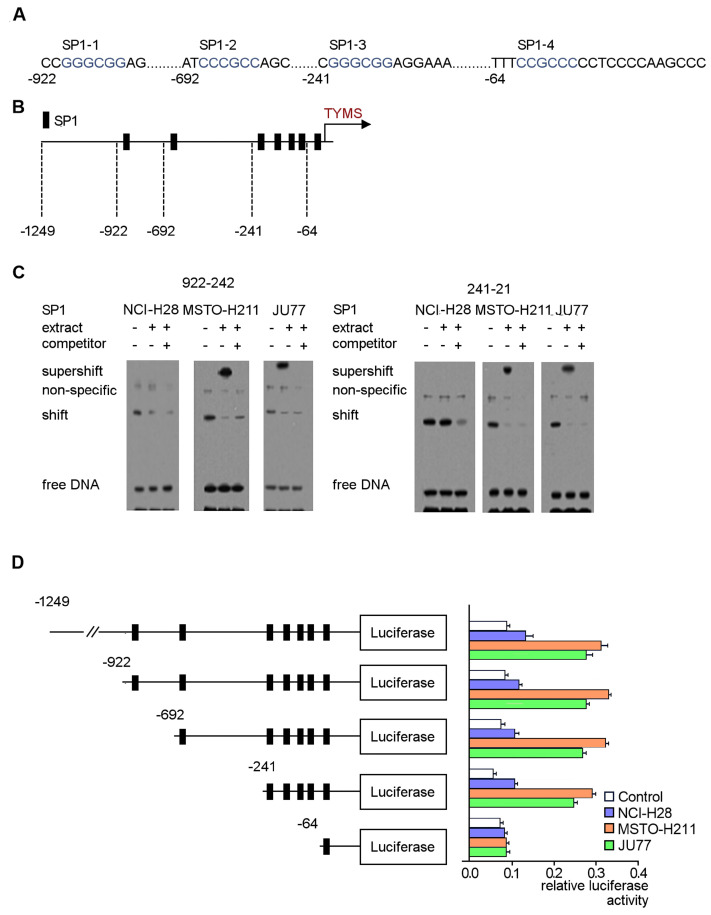
Binding of SP1 nuclear factor to the human *tyms* promoter SP1-specific motifs (**A**). The fragment of the *tyms* promoter, where the SP1 domain (blue) was marked based on Genomatix analysis. (**B**). Schematic map of the human *tyms* promoter fragment (−1249/*tyms*) (red) with potential SP1 binding motifs. (**C**) Nuclear extracts isolated from MSTO-H211, JU77 and control NCI-H28 cell lines used for the EMSA experiment. (**D**) The *tyms*–dual luciferase constructs studied in MSTO-H211, JU77 and control NCI-H28 cells are as follows: total −1249/−21, −922/−21, −692/−21, −241/−21 and −64/−21. The results are the mean of the relative luciferase activity signal (*n* = 3) ± SD. The uncropped blots are shown in Supplementary File S1.

Because SP1 regulates the basal activity of the *tyms* promoter [17] we studied the SP1-responsive regions on the *tyms* promoter in mesothelioma cells. The in silico analyses identified the four most predictable, possible GC-box motifs and selected for further SP1-responsive element investigation to determine which were SP1 bound: −922 to −242 and −241 to −21 (Figure 6A,B). Specific anti-SP1 labeling confirmed the unique SP1 interaction location on the *tyms* promoter in MSTO-H211 and JU77 cells (lane 2 in each scan; Figure 6C). No supershift formation was detected in the NCI-H28 cells.

To determine which particular SP1 motif is responsible for the direct regulation of the *TYMS* expression, we constructed selective 5′ promoter deletants which eliminated the SP1-4 binding motif, as cells transfected with the −62/*tyms* promoter could not express *tyms* in a luciferase reporter assay. However, deletion of the SP1–3 sequence (−241/*tyms*) resulted in lower *TYMS* expression, indicating that the two regions are critical for *TYMS* expression on the studied constructs (Figure 6D).

**Figure 7:**
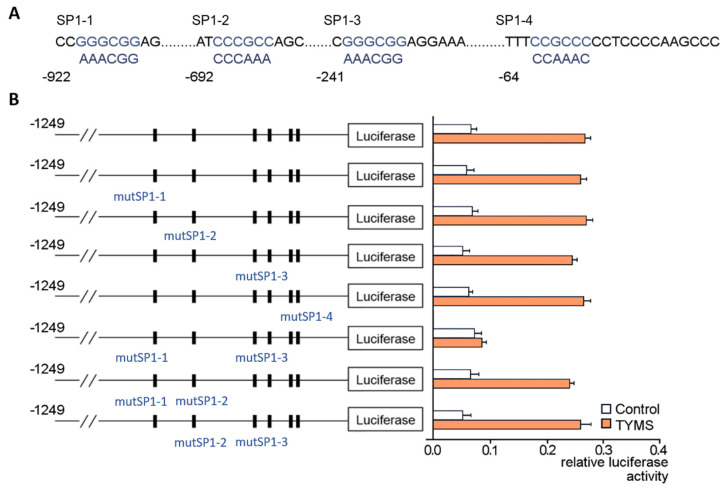
Role of SP1 motifs in SP1-dependent *tyms* expression in MSTO-H211 cells. (**A**) Sequences of mutated SP1-binding sites (bottom dark blue) compared with SP1 motifs (upper light blue) localized on the human *tyms* promoter. (**B**) The *tyms*–dual luciferase constructs studied in MSTO-H211 cells as follows: total −922mutSP1-1/*tyms*, −692mutSP1-2/ *tyms*, −241mutSP1-3/*tyms*, −64mutSP1-4/*tyms* and their mutant combinations. The results are the mean of the relative luciferase activity signal (*n* = 3) ± SD.

Further introduced mutations within the selected SP1 motifs (Figure 7A) resulted in lower transcriptional activity of *TYMS*, where both individual SP1-1 and SP1-3 sites were simultaneously changed (Figure 7B). This indicates the sufficiency of SP1 binding to individual SP1-1 or SP1-3 sites in maintaining *TYMS* expression.

### 3.6. EPE Components Diminish TYMS Expression via SP1-Specific Motifs

**Figure 8:**
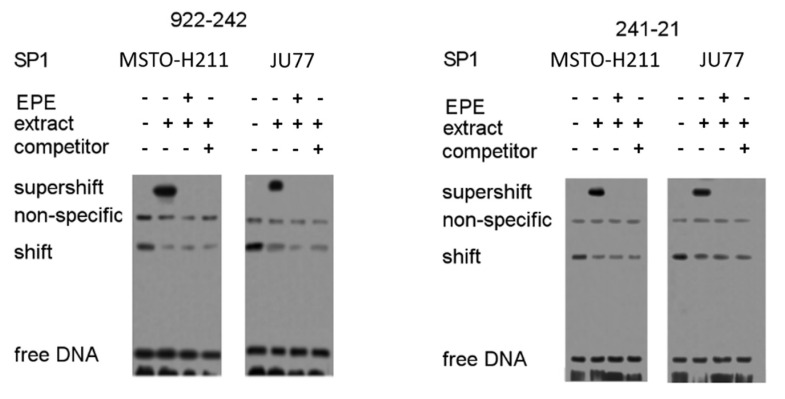
EPE mixture modulates TYMS expression by binding to specific SP1 motifs on the *tyms* promotor. The invasive MSTO-H211 and JU77 cells were treated for 48 h with EPE (200 µg/mL) before the EMSA assay. Nuclear extracts isolated from EPE-treated cells and untreated controls were used for the EMSA experiment. Two separate *tyms* promoter fragments, (−922/−242) and (−241/−21), were investigated. For the competitor test, an unlabeled competitor oligonucleotide was used in excess. For supershift detection, anti-SP1 antibodies were used. The uncropped blots are shown in Supplementary File S1.

The above-presented studies demonstrated that TYMS expression is highly dependent on SP1 modulation. In light of the inhibitory effect of single polyphenol compounds on SP1 activity [7] and the above-demonstrated decreased mRNA and protein levels of TYMS in JU77 and MSTO-H211 cells upon EPE treatment (Figure 4A), we decided to examine the mechanism of TYMS reduction. Based on EMSA analysis, we found that the binding of SP1 to the *tyms* promoter construct region of −241 to −21 (SP1-3, SP1-4) was inhibited by EPE components in invasive MPM cells compared with untreated controls, suggesting a direct blocking of the SP1-3 motif by EPE mixture compounds (Figure 8).

**Figure 9:**
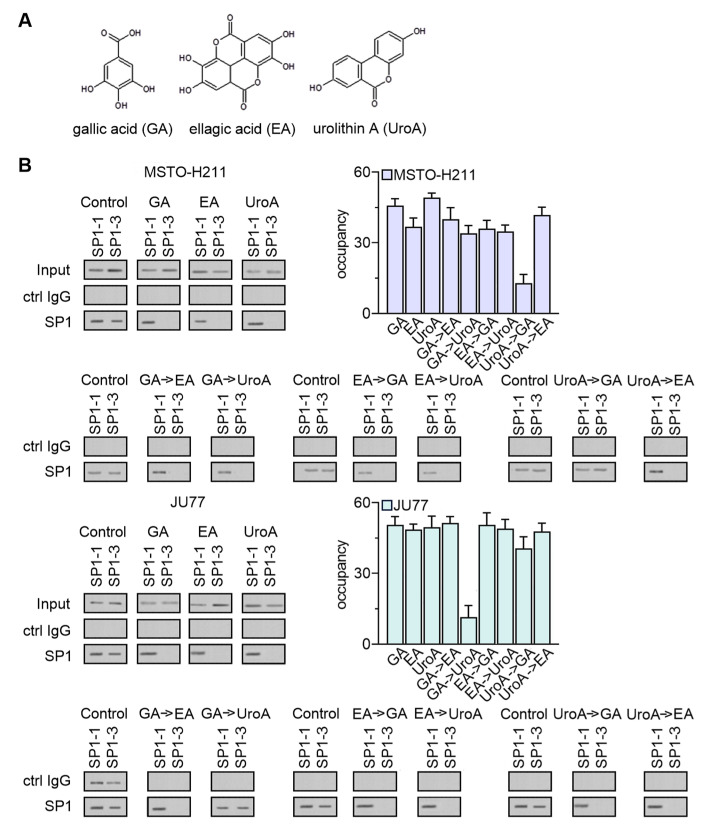
Inhibitory effect of EPE components on *tyms*-promoter-specific SP1 motifs. (**A**) Chemical structures of gallic acid (GA), ellagic and (EA) and urolithin A (UroA) drawn in Chemsketch version ACD/Labs 2023.1.0 software. (**B**) ChIP Assay in MSTO-H211 and JU77 cells after 48 h of treatment with GA, EA and UroA at 41.6 µg/mL GAE concentration. The experiments on fragmented chromatin were carried out with normal rabbit IgG and anti-SP1 antibodies. For the Re-ChIP assay, immunoprecipitated DNA was diluted 10 times and re-immunoprecipitated with normal rabbit IgG and SP1 antibodies. Immunoprecipitated and re-immunoprecipitated DNA was amplified by PCR, separated on polyacrylamide gel, and visualized. Representative inverted gel images are shown. The results are the means ± SD (*n* = 3). The uncropped blots are shown in Supplementary File S1.

Next, we selected reference compounds (with the identified ability to block cell growth and invasion) and verified *tyms* promoter inhibitory effect of single EPE components: EA and GA, and UroA as the EA metabolite [28] (Figure 9A). Our studies demonstrated that each tested compound might interact with the SP1-3 on the *tyms* promoter and partially inhibit its expression (Figure 9B). While the effect of ellagic acid was powerful, the other compounds inhibited the process by the different SP1 motifs, suggesting weaker participation in *TYMS* expression downregulation. Interestingly, the effect of UroA was much more considerable in MSTO-H211. In contrast, GA showed a higher affinity for blocking *TYMS* expression in the JU77 line, implying the involvement of EPE components in different MPM phenotypes.

To confirm the last observation, we used the sequential chromatin immunoprecipitation (SeqChIP) assay, according to the Geisberg method [23], with modifications [29] to distinguish between two molecules that might occupy the same place on the *tyms* promoter. We confirmed that GA preferentially binds to the SP1-3 even in the presence of EA and UroA (Figure 9B), while in the JU77 cells the highest affinity to the *tyms* promoter was achieved by UroA. Therefore, GA is critical in that process in MSTO-H211 cells, whereas UroA is essential in *TYMS* expression in JU77 cells. We hypothesized that applying polyphenol mixtures, such as those in EPE, offers the most significant benefit in regulating *TYMS* expression, regardless of cellular preference.

## 4. Discussion

Due to its long latency period, malignant pleural mesothelioma (MPM) is typically recognized at an advanced stage, resulting in poor patient prognosis. As the tumor is highly aggressive, eliminating its metastatic features is critical for MPM therapy. Thymidylate synthase (TYMS) is elevated in numerous tumors [30,31,32] and its overexpression corresponds to worsening clinical outcomes and has been correlated with drug resistance in malignant cells, including mesothelioma patients [3,5,33,34].

In the present study, we correlated TYMS expression with the previously reported invasive capacity of particular MPM cell lines (a correlation coefficient 0.84) [14] as TYMS expression corresponds with the stage of cancer development and epithelial-to-mesenchymal transition process [4,35,36,37]. TYMS has been described by Fu et al. as a diagnostic and prognostic marker, whose expression increases in the later stages of pancreatic cancer [37]. Strong dependence has also been observed in the hepatocellular carcinoma and metastasis colorectal cancer, serving as a predictive marker of poor outcomes in CRC patients [35,38]. A similar observation was made by Jiang et al. [36], who confirmed the high predictive value of TYMS when evaluating the efficiency of chemotherapy. The authors observed an improved 3-year survival rate in CRC patients with lower TYMS expression status. Similarly, low TYMS protein level has been recognized by Righi et al. for its predictive value in terms of improved time to progression and overall survival in MPM patients treated with pemetrexed [39]. Because TYMS mRNA expression alone did not influence outcomes of MPM patients treated with pemetrexed [40], under bimodal chemotherapy, and in conjugation with platinum, high TYMS protein expression positively correlated with MPM progression and shorter overall survival. This is in contrast with folylpoly–glutamate synthase protein level (endogenous pemetrexed converting enzyme) [41]. Other studies revealed a tumor-stage-dependent upregulation of TYMS in colon cancer cell lines [42]. Additionally, similar results have been recently reported by our group [4] in studies wherein TYMS overexpression was strongly correlated with the maintenance of the EMT process, similar to the breast and lung tumors [4,30,43]. In the present study, we confirmed the positive association of overexpressed *TYMS* with upregulated *TWIST1* and N-cadherin mesenchymal markers, whose expressions demonstrate a predisposition toward extracellular matrix degradation and further tumor invasion and metastasis [44]. In addition, *TWIST1* has been associated with increased N-cadherin regulation and a diminished epithelial biomarker, E-cadherin, and has the features seen in our transcriptomic data analysis. Our in vitro results are consistent with the global transcriptomic data set analysis [45], as we confirmed that invasive biphasic and sarcomatoid MPM cancer cells belong to neoplasms with overexpressed TYMS level, in turn predisposing them to targeted treatment that is accomplished by direct TYMS downregulation, including hampered transcription.

Polyphenols are potent bioactive compounds in cancer management due to their documented modulatory effects on molecular pathways directly involved in carcinogenesis [46], including induction of apoptotic signals, regulation of tumor suppressor genes and oncogenes and stimulation of immune defense against cancer [47]. We have previously reported that a non-cytotoxic concentration of evening primrose extract (EPE) inhibits the invasion and proliferation of MPM and CRC cells [9,14]. Herein, we have confirmed that TYMS is sufficiently downregulated in invasive MPM cells in response to EPE. The administration of individual EPE compounds, gallic acid and ellagic acid, showed a weaker TYMS expression regulation than the EPE preparation, demonstrating the beneficial effect of complex polyphenol mixture over the use of single phytochemicals in invasive neoplasm treatment. Because decreasing TYMS in response to EPE diversifies the effect of EMT in more invasive CRC cancer cells [9], the previously reported diminished mobility and invasive ability of MPM cells upon EPE treatment [14] is accomplished with TYMS downregulation as well.

Specificity protein 1 (SP1) belongs to nuclear factors engaged in the modulation of multiple genes involved in the migration and invasion processes of malignancies [18,48], including TYMS expression regulation [17,49]. Our bioinformatics analysis on clinical transcriptomic data sets and in vitro studies did not reveal the significant difference in SP1 levels observed by others, where high expression of SP1 was notably correlated with the presence of mesenchymal markers such as Snail, L1CAM and VIM, and a reduced epithelial cadherin [50]. However, in clinical studies, the authors did not confirm the significant interdependence of overexpressed SP1 status with lymph node metastasis and progressive invasion in pancreatic adenocarcinoma patients. Still, the silencing of SP1 seems to be a promising target in cancer treatment due to the observed marked decrease in cell motility and invasiveness of tumors [51,52]. Treatment of prostate cancer cells with mithramycin A (MitA), a natural SP1 inhibitor, led to the inhibition of EMT invasive markers and Wnt/β-cadherin signaling, alternating the metastatic ability of cells [53]. Targeting SP1 function by the strategy of silencing either MitA or siRNA disrupted the neovascularization of aggressive subtype [54]. Because SP1 acts on gene promotor regions associated with other transcription factors, depletion of SP1 potentially reduced the interaction with twist in vascular endothelial cadherin expression in prostate cancer cells [54]. Despite the indicated correlation of SP1 with EMT markers in the neoplasms mentioned above, our analysis performed on the clinical data of MPM tumor samples did not confirm this phenomenon. Our Pearson matrix results demonstrate a markedly negative correlation of *SP1* with *TWIST1* mesenchymal biomarkers in MPM individuals. However, it is worth mentioning that heterogeneous material obtained from patients and the complex activity of SP1 in regulating numerous target genes might complicate our results, especially as the SP1 signaling pathway is not orphaned in EMT modeling. The analysis of cell lines confirmed that they were characterized by cells of higher invasion ability (JU77 and MSTO-H211) in contrast with the low invasive NCI-H28. Our observations were consistent with previous studies in which a high level of N-cadherin and a low level of E-cadherin in MSTO-H211 cells were observed [55,56]. Additionally, we revealed that EPE treatment resulted in the downregulation of Snail and ZEB-1—two crucial transcription factors engaged in EMT regulation—as well as N-cadherin and vimentin, the main mesenchymal markers [57,58]. Similarly, epithelial marker levels of E-cadherin and occludin increased. This observation is consist with our previous study conducted on CRC models, in which we observed that EPE reversed EMT phenotype [9]. Others have reported that SP1 is sufficiently downregulated in response to natural compound treatment, including in MPM studies [7,8,59]. Utilization of an SP1 knockdown strategy inhibited the growth, migration and clonogenicity of MPM cells [60]. In our study, we observed a comparable expression of SP1 in all of the studied MPM cells, which is in line with our clinical MPM data analysis. Both of the performed EPE and individual EPE compound treatments established non-significantly reduced SP1 protein level in examined in vitro MPM models. Intriguingly, negatively regulated SP1 translocation into the nucleus in cells with more invasive and migrative phenotypes was observed. Because the SP1 translocation into the nucleus is tailored by the direct interaction of the SP1 molecule with the endogenous importin α transporter [61], and is modulated in response to environmental tumor factors [62], we suggest a possible protein–polyphenol interaction with the SP1 nuclear factor and/or the direct occupation of the *tyms* promoter by EPE-delivered compounds. We hypothesized that there is engagement of EPE components in SP1-dependent *TYMS* expression modulation and sought to analyze the molecular mechanisms of the regulatory activity of EPE and its single ingredients at the transcriptional level.

Considering the critical role of SP1 in regulating TYMS expression [17] and the engagement of both in the metastasis and invasion of severe tumors, including MPM, we investigated the mechanism of TYMS downregulation by EPE. The in silico *tyms* promoter study confirmed the presence of two putative SP1 motifs (SP1-1 and SP1-3) promoting *tyms* overexpression, with a critical role for the SP1–3 sequence in reduced *tyms* promoter activity. Until today, no data have been available on the direct occupation of *tyms* promoters by single or complex chemical structures. The confirmed lack of SP1 interaction with SP1–3, containing sequence −241/−21, on the *tyms* promoter, is consistent with mutational deletants of SP1-binding motifs. Our results agree with the previously demonstrated putative SP1 binding motif on *tyms* promoter in MCF-7 breast cancer cells, wherein the regions −229/−140 and −145/−124 were investigated and found to be responsible for the high basal activity of TYMS [63]. Additionally, the latter *tyms* sequence is conserved between species, as indicated by Rudge et al. [64]. In conclusion, the SP1 motif located in the nearest fragment to the *tyms* transcription start site, described as an essential regulator of *tyms* expression, is especially important in the EPE inhibitory effect.

Our results challenge the idea of the natural complex use in cancer treatment rather than individual EPE compounds intervention. The co-occupancy study confirmed that single reference standards interact with *tyms* promoter regions and are directly engaged in SP1-3 region binding, notably in the invasive MPM subtypes. Considering the chemical structure of ellagic acid and its metabolite—urolithin A—direct intercalation of these molecules into the DNA structure bearing SP1-specific motifs on the *tyms* promoter sequence is possible. Because each compound showed different SP1 element binding specificities, treating MPM cancer cells with a defined EPE mixture seems more reasonable. Our observation aligns with others who postulate the combinatory use of natural compounds, rather than a single administration of a desired chemical [65,66]. Considering the above, the phytochemicals present in the EPE preparation exhibit bifunctional activity in both MPM cancer cells’ cellular and nuclear compartments, including modulatory effects on downstream signaling factors and direct transcription regulation.

## 5. Conclusions

In conclusion, for the first time we have delineated the transcriptional relationship of the oncogenic SP1 transcription factor in the human *tyms* promoter’s anticancer activity of EPE isolated from *Oenothera paradoxa* seeds. Our results strongly suggest a significant benefit from the use of polyphenol-rich extracts, such as EPE, over individual compound intervention, when triggering thymidylate synthase, one of the prevailing targets in oncology, including in the treatment of MPM tumors. The established natural EPE delivers a synergistic effect when suppressing SP1 activity and SP1-related gene transcription, allowing one to simultaneously avoid both cell and tissue specificity regarding sensitivity to individual polyphenols. To fully resolve the interaction between protein and polyphenols, further in-depth analysis of the molecular docking of polyphenol compounds involved in MPM growth suppression is required.

## Figures and Tables

**Table 1:** The sequences of the forward and reverse primers used in the study.

Gene	Primers 5′-3′		Amplicon Length
	Forward	Reverse	
*TYMS*	CGCTACAGCCTGAGAGATGAA	CACTCCCTTGGAAGACAGCTC	129 bp
*SP1*	AGTGTATGGCAAGACCTCTCAC	TCTTCTCACCTGTGTGTGTACG	149 bp
GAPDH *	GGTGGTCTCCTCTGACTTCAACA	GTTGCTGTAGCCAAATTCGTTGT	127 bp

* Reference gene.

## Data Availability

The data presented in this study are available on request from the corresponding author.

## Supplementary Materials

The following supporting information can be downloaded at: https://www.mdpi.com/article/10.3390/cancers15205003/s1, Figure S1. Invasion ability increases in more invasive MPM cell lines. Supplementary File S1: Uncropped blots.
