# Supplementary material for: Evening Primrose Extract Modulates TYMS Expression via SP1 Transcription Factor in Malignant Pleural Mesothelioma
Source: Cancers (Basel). 2023 Oct 16;15(20):5003. doi: 10.3390/cancers15205003 (PMC10605291; doi:10.3390/cancers15205003)
Supplement: Supplementary file 1 [file cancers-15-05003-s001.zip › Supplementary Figure S1.pdf]

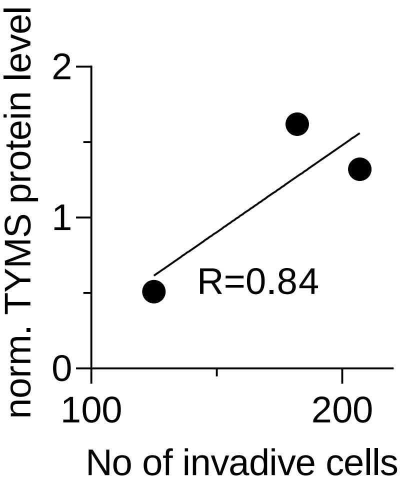

Figure S1. Invasion ability increases in more invasive MPM cell lines. The observed in previous studies invasion ability of MSTO-H211, JU77 and NCI-H28 cell lines [14] were correlated with TYMS protein level. The correlation coefficient (R)between invaded cells and TYMS protein level, calculated in GraphPad Software, amounted to 0.84.
