# Supplementary material for: Evening Primrose Extract Modulates TYMS Expression via SP1 Transcription Factor in Malignant Pleural Mesothelioma
Source: Cancers (Basel). 2023 Oct 16;15(20):5003. doi: 10.3390/cancers15205003 (PMC10605291; doi:10.3390/cancers15205003)

blots Figure 3

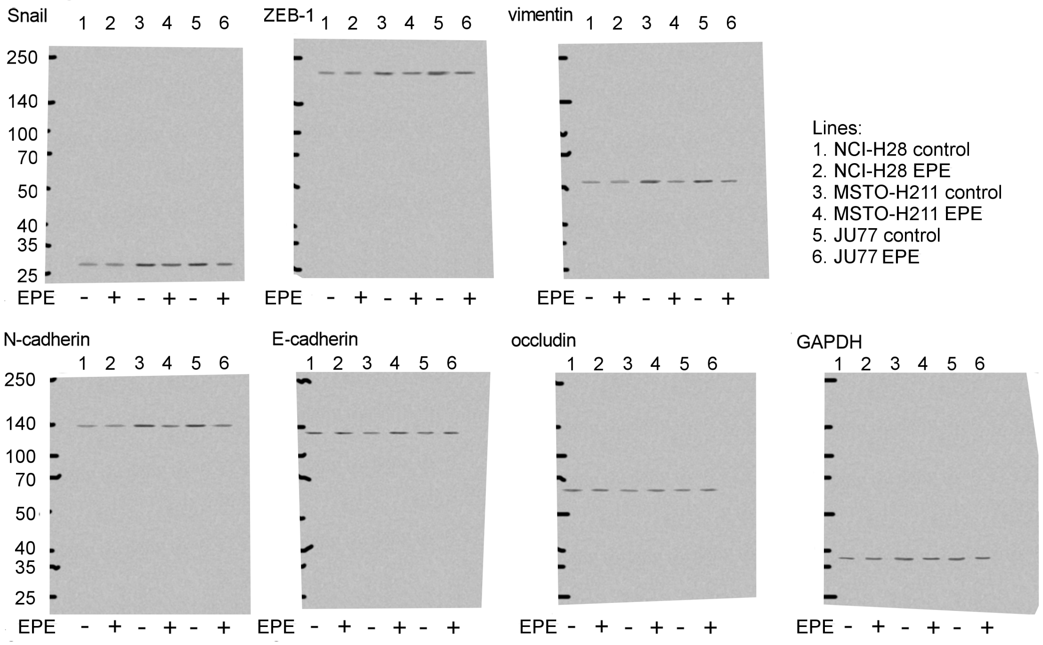

blots Figure 4

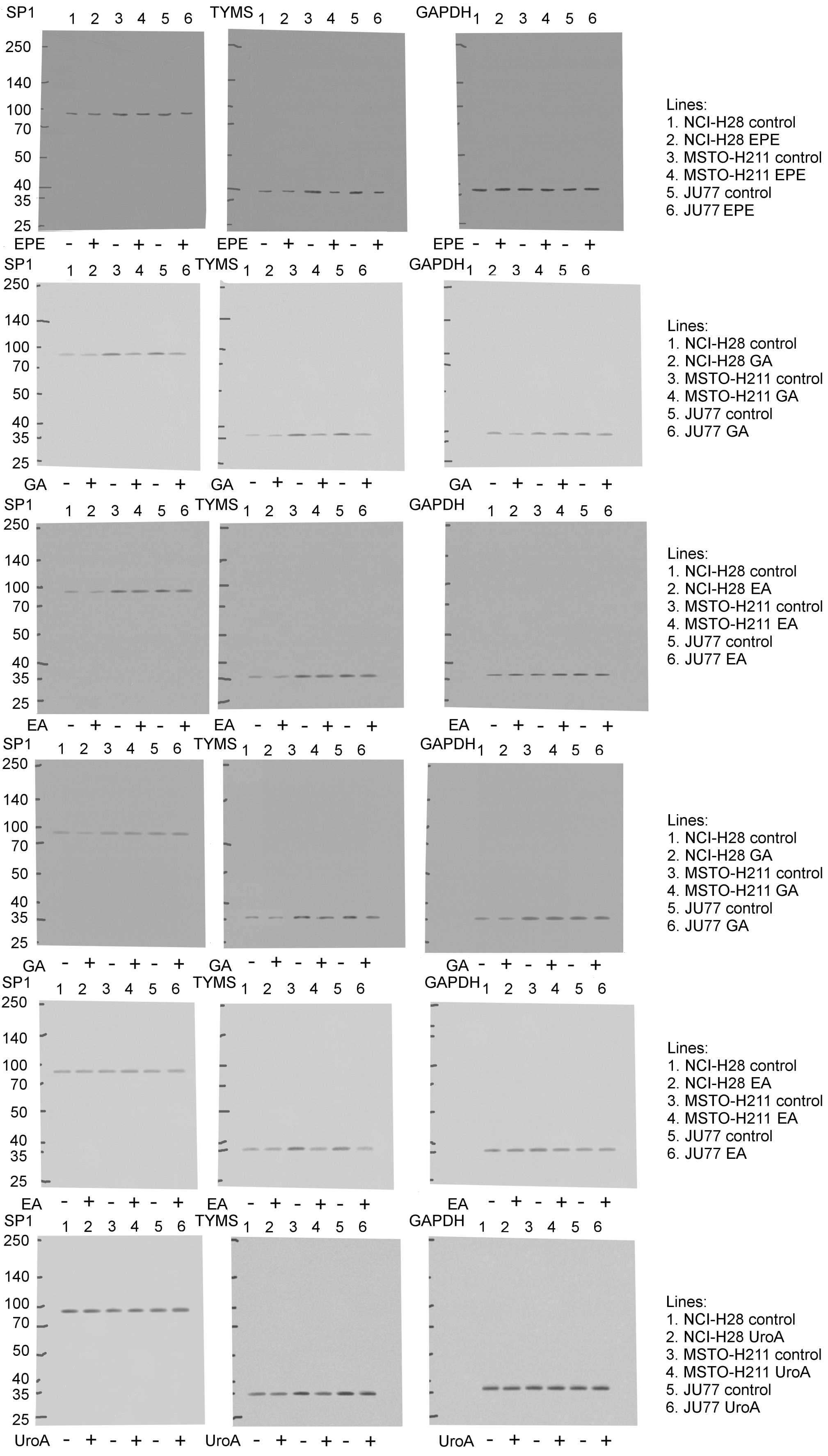

blots Figure 5

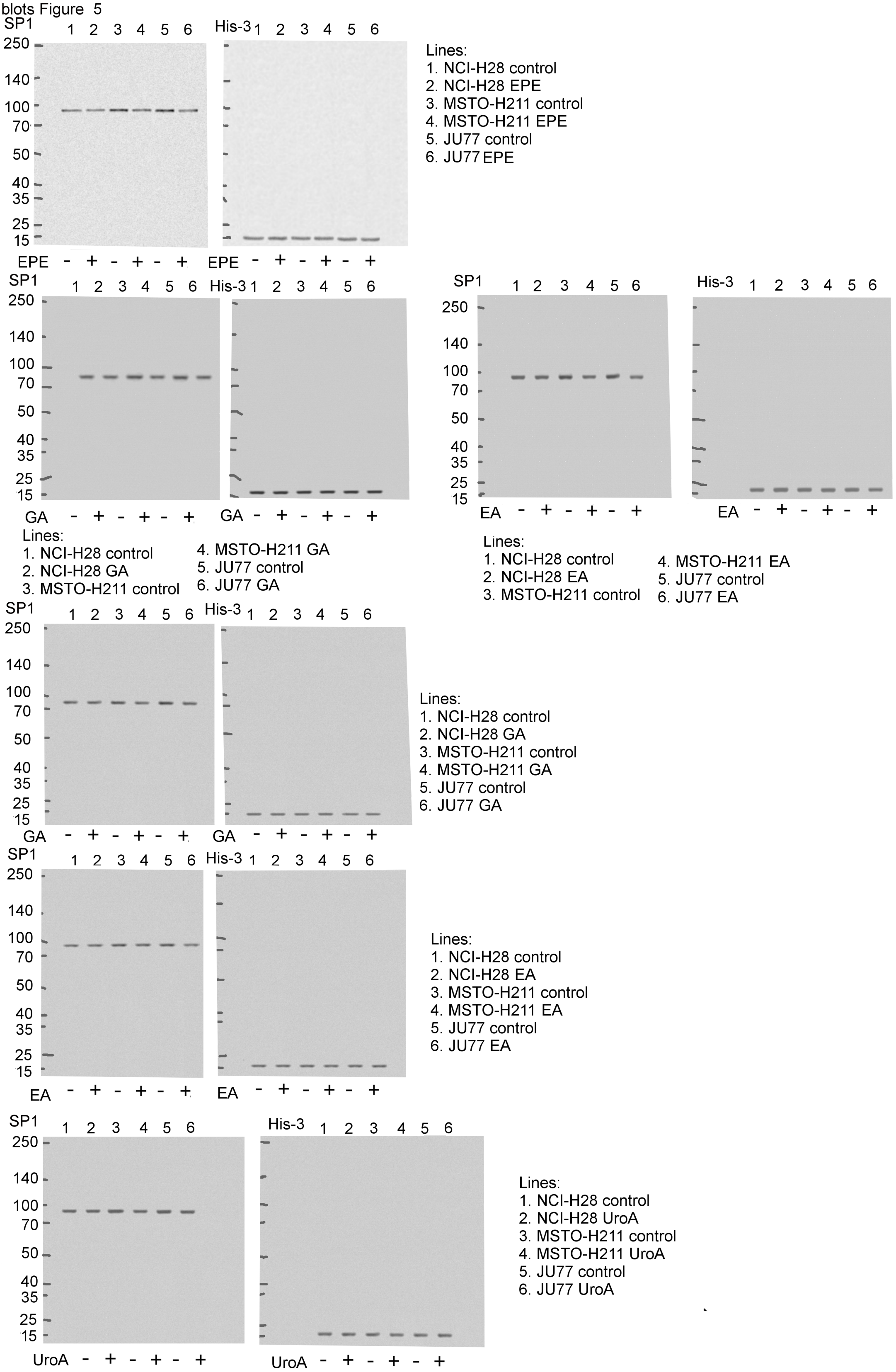

blots Figure 6

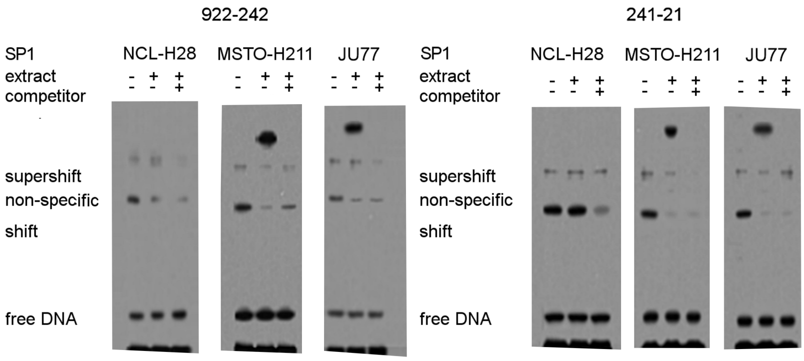

blots Figure 8

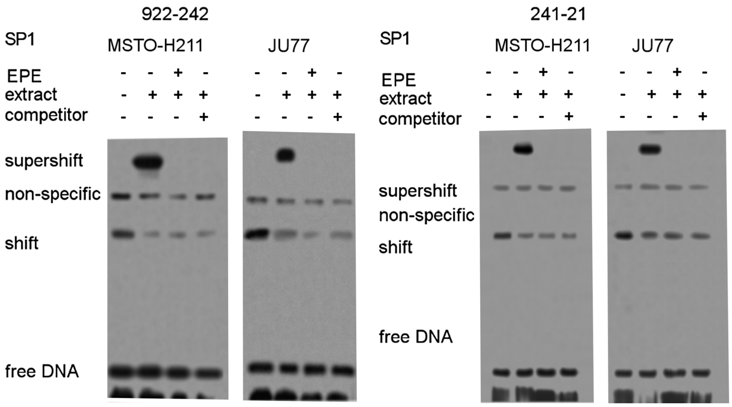

blots Figure 9
SP1

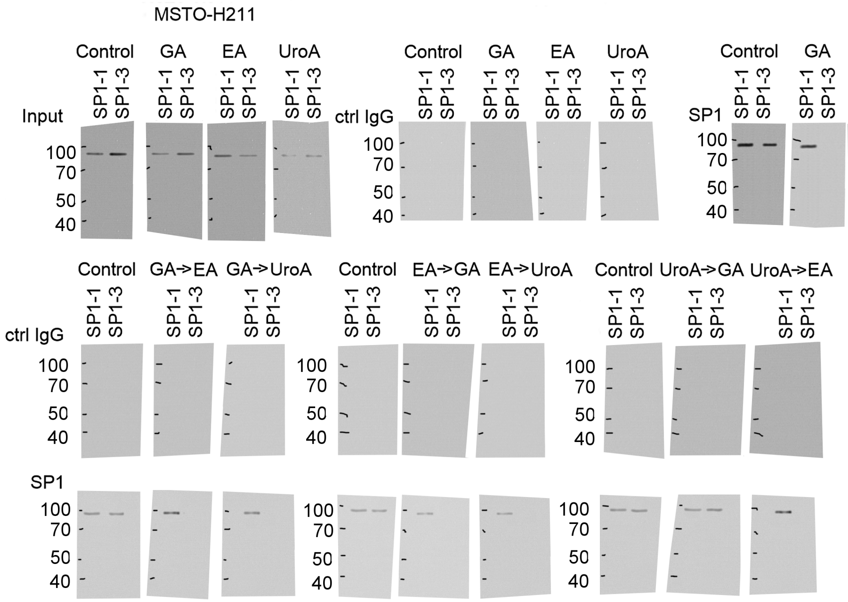

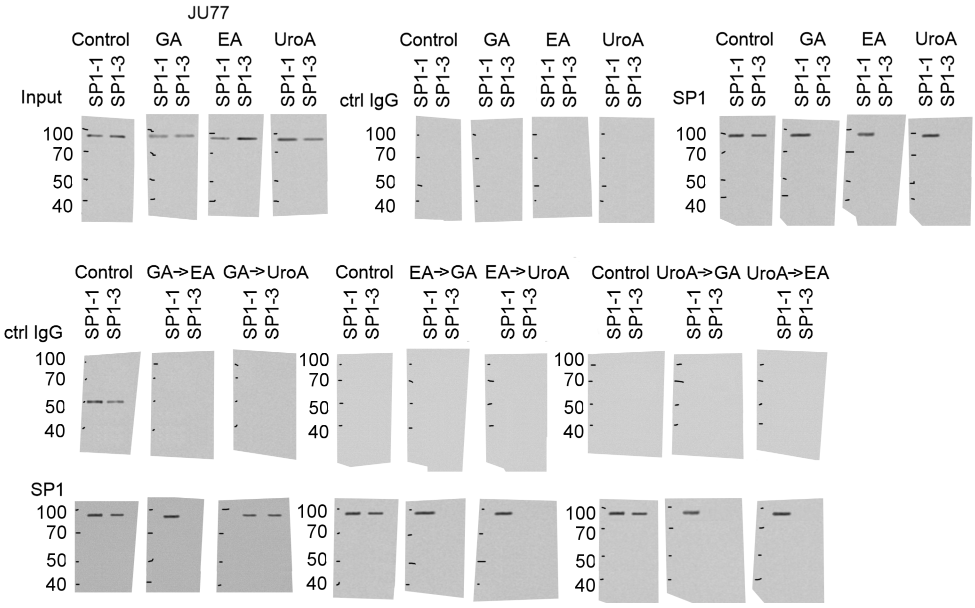

Supplement: Supplementary file 1 [file cancers-15-05003-s001.zip › Supplementary File S1 uncropped blots.pdf]
